# Common Iliac Vein Injury in Blunt Abdominal Trauma Without Pelvic Fractures

**DOI:** 10.7759/cureus.14401

**Published:** 2021-04-10

**Authors:** Nicholas W Sheets, Olivas Roderick, David S Plurad

**Affiliations:** 1 Trauma and Acute Care Surgery, Riverside Community Hospital, Riverside, USA; 2 General Surgery, Riverside Community Hospital, Riverside, USA

**Keywords:** iliac vein, trauma imaging, trauma, blunt trauma, pelvic fracture, iliac, lumbar hernia, hematoma, vascular trauma, case study

## Abstract

Iliac vein injury in the absence of pelvic fractures is rare. We present the case of a 27-year-old male involved in a motorcycle crash. Imaging demonstrated a lumbar hernia and pelvic hematoma in the absence of pelvic fractures. The patient became unstable and required emergency surgery demonstrating an iliac vein injury requiring ligation. Diagnosis and management of this rare injury is reviewed.

## Introduction

Iliac vascular injuries occur in 2.3% of abdominal trauma patients and are associated with a 39% mortality rate [1]. The presence of an iliac vein injury in the absence of pelvic fractures is a rare finding [2,3]. Isolated iliac vein injuries may result from stretch injuries leading to retroperitoneal hematomas prompting the diagnosis [2]. The diagnosis and management of this rare injury is complex. We present a case of a blunt trauma patient without pelvic fractures who was diagnosed with an iliac vein injury and discuss the diagnosis and management.

## Case presentation

**Figure 1:**
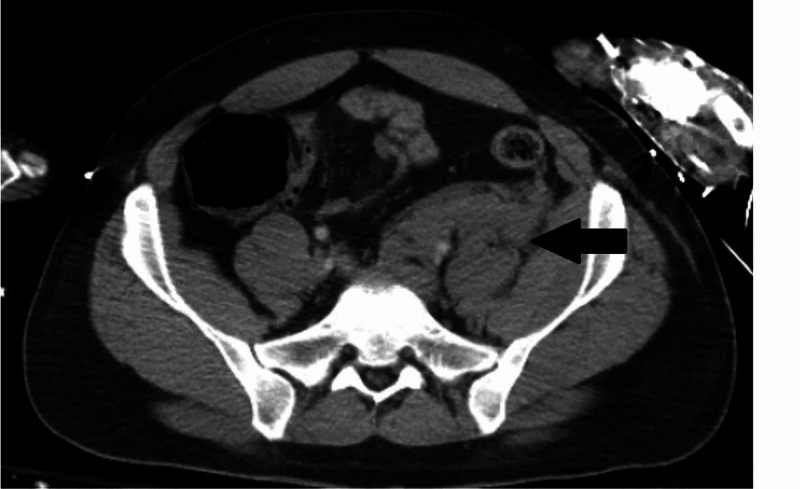
Pelvic hematoma without pelvic fractures.

**Figure 2:**
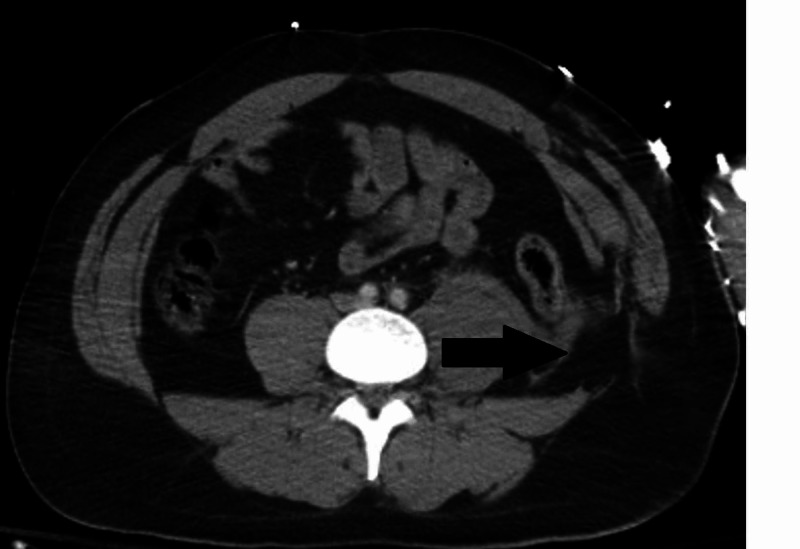
Traumatic left lumbar hernia.

A 27-year-old male presented after a motorcycle versus semi-trailer truck at freeway speed. He complained of left thigh and abdominal pain. The patient had no comorbidities and presented with Glasgow Coma Scale score 15 and was hemodynamically stable. Radiographs demonstrated a comminuted displaced and bayoneted acute left femoral subtrochanteric fracture with extension into the lesser femoral trochanter and a complex left ankle fracture. A full computed tomography (CT) scan with contrast demonstrated a grade 2 liver laceration, grade 3 left kidney laceration, bilateral first rib and scapula fractures, a left pelvic retroperitoneal hematoma with hemoperitoneum (Figure 1) and a traumatic left lumbar hernia (Figure 2). Shortly afterwards the patient became hypotensive and developed peritonitis; therefore, massive transfusion protocol was initiated and the patient was transferred to the operating room. On exploration, a left-sided traumatic abdominal wall hernia was identified with significant bleeding. The left colon was medialized exposing the abdominal wall hernia and the left iliac vessels. A full-length longitudinal tear along the anterior aspect of the left common iliac vein was noted with significant bleeding. The left hypogastric artery was ligated, transected, and mobilized to expose the full extent of the injury. Distal and proximal digital control was achieved, and the vein was ligated. There was no further bleeding and hemostasis was obtained. Total estimated blood loss was 2,000 cc. The remainder of the abdomen was inspected and did not require intervention. The abdomen remained open postoperatively due to massive resuscitation resulting in coagulopathy and hypothermia. The abdomen was closed on the following day and the traumatic hernia left in situ for potential future repair due to his concurrent injuries. His multiple orthopedic injuries were repaired and he received surgical debridement of a thigh, pelvic, and arm degloving injury. He was diagnosed with a left femoral deep vein thrombosis (DVT) on screening ultrasound and received therapeutic anticoagulation. The patient had a 28-day hospital stay and was discharged to a rehabilitation facility.

## Discussion

An iliac vein injury in the absence of pelvic fractures is rare with few reported cases. In a review of the National Trauma Data Bank, isolated iliac vein injuries occur in 0.1% of all abdominal blunt trauma patients with a 16.5% 30-day mortality [1]. The diagnosis of the isolated iliac vein injuries can be difficult. In our case, as in previous reports, the presence of a retroperitoneal hematoma was identified on CT scan [2,3]. In the presented case, a traumatic lumbar hernia was also present likely representative of the high energy mechanism. The diagnosis can be made by either venogram or delayed contrast CT scan in patients that are stable [2,4], or by direct exploration in patients who are unstable or require surgery for other reasons [3]. The management of iliac vein injuries is usually mandated by several injury and patient factors. The decision of ligation versus primary repair remains controversial, while endovascular repair with uncovered and covered stents has been described [1,4,5]. In the case presented by Boulanger et al., a longitudinal injury was identified that was amenable to primary repair in the hemodynamically stable patient yet treated with ligation in the presented case due to instability needing massive transfusion protocol [3]. The most common reported morbidity after iliac vein injuries are DVT in 12.6% of patients, followed by the need for fasciotomy in 11.5% of patients [1]. Outcomes regarding ligation versus primary repair are not statistically different regarding DVT, pulmonary embolism, or fasciotomy, yet there is a higher 30-day mortality associated with ligation (18.8%) versus primary repair (8.8%) [1].

## Conclusions

Iliac vein injuries should be suspected in blunt trauma patients with retroperitoneal hematomas despite the absence of pelvic fractures. Additional findings of a blunt high energy mechanism such as traumatic lumbar hernias should prompt high clinical suspicion. Diagnostic modalities include venogram, delayed contrast CT scan, or surgical exploration. While primary repair or ligation remains a viable option, endovascular treatment should remain in the surgeon’s armamentarium. The associated morbidity and mortality of this rare injury remains significant requiring prompt recognition and treatment.
